# The YEATS family member GAS41 interacts with the general transcription factor TFIIF

**DOI:** 10.1186/1471-2199-11-53

**Published:** 2010-07-12

**Authors:** Sabrina Heisel, Nunja C Habel, Nicole Schuetz, Alessia Ruggieri, Eckart Meese

**Affiliations:** 1 Department of Human Genetics, Saarland University, 66421 Homburg, Germany; 2 Department of Infectious Diseases, Molecular Virology, Medical Faculty of Heidelberg, 69120 Heidelberg, Germany

## Abstract

**Background:**

In eukaryotes the transcription initiation by RNA polymerase II requires numerous general and regulatory factors including general transcription factors. The general transcription factor TFIIF controls the activity of the RNA polymerase II both at the initiation and elongation stages. The glioma amplified sequence 41 (GAS41) has been associated with TFIIF via its YEATS domain.

**Results:**

Using GST pull-down assays, we demonstrated that GAS41 binds to both, the small subunit (RAP30) and the large subunit (RAP74) of TFIIF *in vitro*. The *in vivo *interaction of GAS41 and endogenous RAP30 and RAP74 was confirmed by co-immunoprecipitation. GAS41 binds to two non-overlapping regions of the C-terminus of RAP30. There is also an ionic component to the binding between GAS41 and RAP30. There was no evidence for a direct interaction between GAS41 and TBP or between GAS41 and RNA polymerase II.

**Conclusions:**

Our results demonstrate binding between endogenous GAS41 and the endogenous TFIIF subunits (RAP30 and RAP74). Since we did not find evidence for a binding of GAS41 to TBP or RNA polymerase II, GAS41 seems to preferentially bind to TFIIF. GAS41 that does not contain a DNA-binding domain appears to be a co-factor of TFIIF.

## Background

In eukaryotes general transcription factors control the activity of RNA polymerase II during initiation and elongation of mRNA synthesis. The transcription initiation requires the concerted action of a complex of transcription factors including TFIIA, TFIIB, TFIID, TFIIE, TFIIF, and TFIIH [1,2]. Regulation of transcription is likely to be concerted by additional regulating factors, which are distinct from the general transcription factors in that they are dispensable for basal transcription. Cofactors may also be distinct, because some of them do not directly bind DNA [3]. Some cofactors seem to bridge the interaction between gene-specific transcription factors and general transcription factors, whereas others facilitate chromatin remodeling [4].

An improved understanding of transcription requires further elucidation of the RNA polymerase II machinery. Several lines of evidence suggest that glioma amplified sequence 41 (GAS41) is associated with the general transcription factor complex. Originally, we isolated GAS41 from a glioblastoma cell line as a nuclear protein containing a C-terminal alpha-acidic activation domain and an N-terminal YEATS domain [5]. This YEATS domain is conserved in the YEATS family of transcription factors, including human AF9, ENL, and the yeast ANC1/Taf14 protein. All of the YEATS proteins are components of multi-subunit complexes involved in transcription regulation and chromatin remodeling [6]. For instance, Taf14 is a subunit of TFIID and TFIIF, the chromatin remodeling complexes SWI/SNF, RSC and INO80 as well as the histone acetyltransferase complex NuA3 [7,8]. GAS41 that shows homology to the N-terminus of Taf14 interacts with INI1, the human homolog of the SWI/SNF complex component SNF5 [9].

Furthermore, GAS41 is a subunit of the human TIP60 and SCRAP complexes [10,11]. Targeted disruption of the *GAS41 *gene in chicken indicates that GAS41 is required for RNA transcription. GAS41 is suggested to function at the assembly of general transcription initiation complexes at the nuclear matrix [12]. We asked whether GAS41 is also associated with the human general transcription factor complex. We examined the interaction of GAS41 and TFIIF which is a heteromeric tetramer of RAP30 and RAP74 [13].

## Results

### Association of GAS41 with the general transcription factor TFIIF

**Figure 1:**
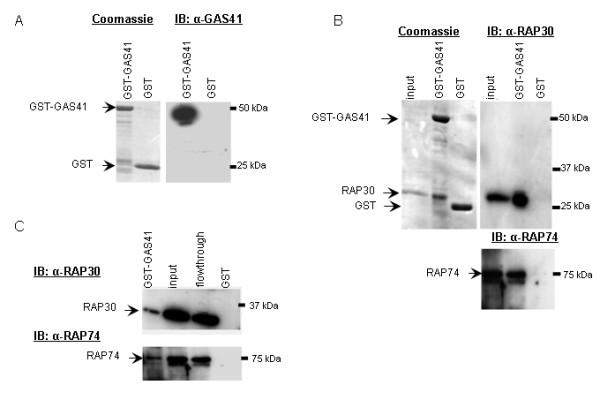
**GAS41 directly interacts with TFIIF**. (A) GST-GAS41 and GST were purified and subjected to SDS-PAGE. Coomassie staining (left) and immunoblot using GAS41 antibody (right), identified GST-GAS41 and GST. (B) GST pull-down assay with GST-GAS41 as bait retained His-RAP30, while negative control GST did not. His-RAP74 was retained by GST-GAS41 as bait, but not by GST as negative control. (C) GST pull-down assay using HeLa nuclear extract confirms the *in vitro *interaction of GST-GAS41 with both, RAP30 and RAP74.

To analyze whether GAS41 interacts with TFIIF, we recombinantly expressed GST-GAS41, purified the fusion protein and confirmed the expression by SDS-PAGE. Coomassie staining showed a ~50 kDa signal corresponding to the combined molecular mass of GST-GAS41. Immunoblot analysis with GAS41 antibody also showed a signal of ~50 kDa (Figure 1A). GST pull-down assay was performed with purified GST-GAS41 immobilized on glutathion-sepharose matrix. GST-GAS41 and GST as control were incubated with purified His-RAP30 and His-RAP74. The eluted protein complexes were subjected to immunoblot analysis. Both, His-RAP30 and His-RAP74 were retained by GST-GAS41 but not by GST alone (Figure 1B). To test for binding of GAS41 with endogenous TFIIF, we performed GST-pull-down assays with HeLa nuclear extract as source for general transcription factors. Immunoblotting confirmed binding of GST-GAS41 with both RAP30 and RAP74 (Figure 1C).

### Binding of GAS41 to TFIIF

**Figure 2:**
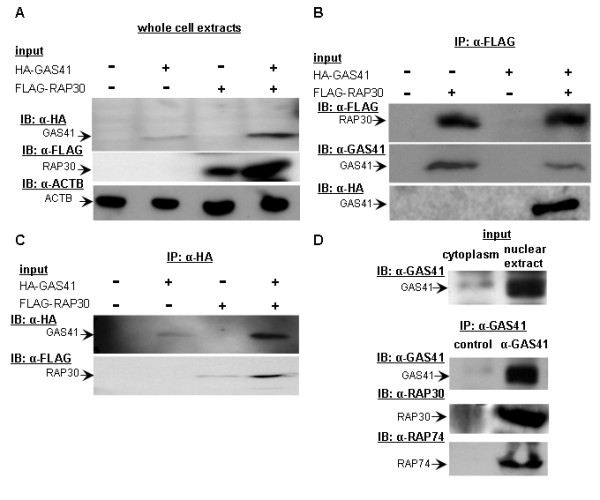
**GAS41 and TFIIF interact *in vivo***. For co-immunoprecipitation HEK293 cells were co-transfected with pSG5-HA-GAS41 and p3xFLAG-CMV10-RAP30. (A) Expression of HA-GAS41 and FLAG-RAP30 was confirmed by immunoblot. (B) Immunoprecipitation of FLAG-RAP30 revealed the *in vivo *interaction of GAS41 and RAP30. (C) Immunoprecipitation of HA-GAS41 and immunoblot using FLAG antibody confirmed this *in vivo *interaction. A weak background signal of FLAG-RAP30 shows in lane 3. (D) Co-immunoprecipitation of endogenous GAS41, RAP30, and RAP74 from HeLa nuclear extracts confirmed the interaction of GAS41 and TFIIF. Cytoplasmic extract was used as negative control (input). For immunoprecipitation, HeLa nuclear extracts were subjected to protein A-sepharose coupled with either GAS41 antibodies or isotype control.

To independently confirm the interaction of GAS41 and TFIIF, we performed co-immunoprecipitation experiments. We transiently co-transfected cells with HA-GAS41 and FLAG-RAP30. Immunoblotting of cell extracts showed co-expression of HA-GAS41 together with FLAG-RAP30 (Figure 2A). Subsequently, cell lysates were incubated either with anti-FLAG-M2 agarose or anti-HA agarose. By FLAG-immunoprecipitation HA-GAS41 as well as endogenous GAS41 was efficiently co-purified in complex with FLAG-RAP30. Vice versa, by HA-immunoprecipitation FLAG-RAP30 was efficiently co-purified in complex with HA-GAS41 (Figure 2B, C).

Additionally, we performed immunoprecipitation experiments with the endogenous, transcriptionally relevant TFIIF complex. Therefore, nuclear extracts from HeLa cells without overexpression of RAP30 or RAP74 were subjected to immunoprecipitation using GAS41 antibodies. Immunoblot analysis using RAP30 and RAP74 antibodies revealed that endogenous GAS41 co-immunoprecipitates with endogenous TFIIF (Figure 2D).

### Binding of GAS41 to TFIIF: Specificity and ionic strength

**Figure 3:**
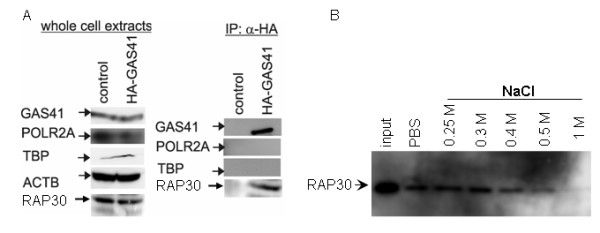
**Specific binding of GAS41 to TFIIF**. HEK293 cells were transfected with pSG5-HA-GAS41 or pSG5. (A) Expression of HA-GAS41, RNA polymerase II, TBP, RAP30, and ACTB was confirmed by immunoblot. Immunoprecipitation of HA-GAS41 and immunoblot disprove a direct interaction of GAS41 and TBP or RNA polymerase II *in vivo*. (B) GST-GAS41 was incubated with HeLa nuclear extract as prey and subjected to extensive washing steps with increasing salt concentration. Immunoblot confirmed the interaction of GST-GAS41 and RAP30 up to 0.5 mM NaCl.

We analyzed the direct interaction of GAS41 and the TATA binding protein (TBP) as well as the RNA polymerase II *in vivo*. Immunoblot analysis revealed no specific binding of TBP to GAS41. Additionally, there is no direct interaction of GAS41 and RNA polymerase II (Figure 3A). These results provide evidence that the GAS41 interaction is specific for this particular general transcription factor.

To examine the stability of protein interactions in the GAS41/TFIIF complex, we analyzed the binding of endogenous RAP30 to the GST-GAS41 fusion protein. GST-GAS41 coupled sepharose was incubated with HeLa nuclear extract. The bound proteins were washed at increasing salt concentrations. Immunoblot analysis showed that NaCl concentrations of up to 0.4 M cause a slight decrease in GAS41-RAP30 interaction. After washing steps with 0.5 M NaCl, RAP30 is only weakly detectable by immunoblot analysis (Figure 3B). These data indicate an ionic component in the interaction between GAS41 and TFIIF.

### Mapping of the GAS41 binding region of RAP30

**Figure 4:**
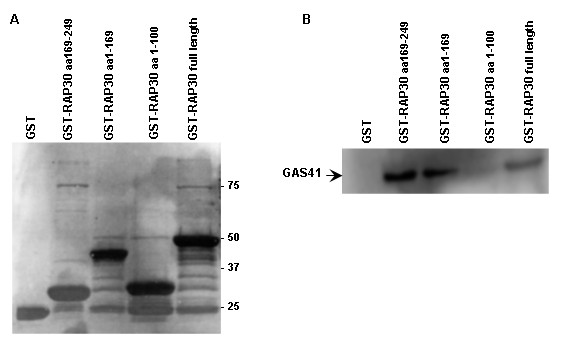
**The GAS41 binding region of RAP30**. The GAS41 binding site of RAP30 was characterized using GST-RAP30 with different protein domains. (A) Coomassie staining demonstrates the expression of RAP30 fragments. (B) GST pull-down assay mapped the GAS41 binding site to the RAP30 fragments d2 and d3.

To delineate the GAS41 binding region of RAP30, we utilized GST-RAP30 truncation mutants. In detail, we generated fusion proteins representing the RAP74 binding domain (d1), the DNA binding site (d3) and a fragment covering the RAP74 binding domain and the polymerase binding domain (d2) [2,13,14]. GST fusion proteins were subjected to pull-down assays using His-GAS41 as prey. Immunoblot analysis revealed the *in vitro *interaction of GAS41 with all constructs but d1. These data indicate that GAS41 interacts with two no-overlapping regions of RAP30 (aa101-169 and aa169-249) covering the polymerase and the DNA binding site (Figure 4).

## Discussion

We described GAS41 as a potential transcription factor containing an activation and a YEATS domain [5,15]. Here, we report GAS41 as a potential cofactor of TFIIF. TFIIF, consisting of the two subunits RAP30 and RAP74 [16-18], inhibits non-specific transcription initiation by recruiting polymerase II to the pre-initiation complex [19,20]. This polymerase II/TFIIF complex attaches through direct interactions of TFIIF with promoter DNA, TFIIB and TFIID [14]. Together with the transcriptional co-activator Mediator, it promotes the association of TFIIE and TFIIH to the pre-initiation complex [21,22].

GST pull-down assays using recombinant RAP30 and RAP74 revealed specific binding of GAS41 to TFIIF *in vitro*. By using HeLa nuclear extract, we confirmed these results by demonstrating that both subunits of endogenous TFIIF interact with GST-GAS41. Additionally, we verified the interaction with RAP30 and RAP74 *in vivo *by co-immunoprecipitation. Since GAS41 does not precipitate TBP or RNA polymerase II, GAS41 appears to preferentially interact with TFIIF. Although, the interaction of GAS41 and RAP30 is resistant to high ionic strength, there is an ionic component to the binding between GAS41 and RAP30.

To further characterize the demonstrated interaction of GAS41 and TFIIF, we expressed full length GST-RAP30 as well as three truncated RAP30 mutants. *In vitro *binding assays revealed direct interaction with all RAP30 constructs except for the RAP74 binding site fragment (d1). We mapped the GAS41 binding site to two non-overlapping regions at the C-terminus of RAP30. Nevertheless, a single GAS41 binding site partly overlapping with the DNA and polymerase binding site can not be excluded. This binding possibly contributes to a function of GAS41 in stabilizing the interaction of TFIIF and DNA.

The function and structure of the RAP30 and RAP74 units have been well defined [14,23-26]. Specifically, RAP30 has been shown to be necessary for pre-initiation complex formation and gene transcription [27-29]. Our data suggest that GAS41 interacts as transcriptional cofactor with the pre-initiation complex via binding to TFIIF. In addition to the recently demonstrated role of GAS41 as co-activator of the sequence-specific transcription factor AP-2β [30] and repressor of the p53 pathway [31,32], the interaction with the pre-initiation complex implies a more general function of GAS41 in cellular transcription. GAS41 is also described as part of TIP60 and SRCAP [32]. Although GAS41 is a component of TIP60, its function in p53 repression is mediated in a TIP60 independent pathway [32]. Therefore, we assume that GAS41 exerts its function in a mechanism which is independent from the chromatin-modifying complexes TIP60 and SRCAP.

The association of GAS41 to the RAP30 C-terminus possibly contributes to a stabilization of the TFIIF-DNA interaction. Although the functional role of GAS41 in transcription remains to be fully elucidated, we suggest that GAS41 as co-factor of the general transcription complex facilitates transcription initiation and bridges the gap to sequence specific transcription by interaction with appropriate transcription factors.

## Conclusion

We demonstrated that endogenous GAS41 directly interacts with endogenous TFIIF. We mapped the GAS41 binding domain to two regions of RAP30 C-terminus. Without *in vivo *evidence for a binding of GAS41 to TBP or RNA polymerase II, GAS41 seems to preferentially bind to TFIIF. Without a DNA-binding domain, GAS41 appears to be a co-factor of TFIIF.

## Methods

### Expression vectors and antibodies

Vector pGEX-4T1 (GE Healthcare) was used to express GST-tagged full length GAS41 and RAP30 in BL21 (DE3), as well as RAP30 fragments d1-d3, covering aa1-100, aa1-169, and aa169-249. His-GAS41 was expressed in M15 [pRep4] (Qiagen), using the pQE30 vector (Qiagen). The plasmids 6HispET11d-RAP30 and 6HispET11d-RAP74 were a kind gift from R.G. Roeder. Vector pSG5 (Stratagene) was used to express HA-GAS41 in HEK293. FLAG-RAP30 was expressed in HEK293 using p3xFLAG-CMV-10 (Sigma). For immunoblot analysis, we used rabbit RAP30 and RAP74 antibodies (Santa Cruz), rabbit anti-GAS41 [15], mouse-anti TBP (Transduction Laboratories), mouse-anti RNA polymerase II (Millipore), goat anti-GST (GE Healthcare), rat anti-HA (Roche) and mouse anti-FLAG- (Sigma). Peroxidase-conjugated antibodies were from Dianova.

### GST pull down assay

GST fusion proteins were expressed in BL21. After induction with 1 mM isopropyl-D-thiogalactopyranoside (IPTG) (3 h, 30°C), cells were sonicated in PBS containing 1% TritonX-100. His-RAP30 and -RAP74 were expressed in BL21 (0.4 mM IPTG, 3 h, 30°C). His-GAS41 was expressed in M15 [pRep4] (0.5 mM IPTG, 2 h, 30°C). Native protein purification by Ni-NTA affinity chromatography was performed according the manufacturer's instruction (Qiagen).

For GST pull-down assay, 10-20 μg GST-fusion protein was coupled to 100 μl glutathione-sepharose 4B (GE Healthcare). Matrix was incubated with 250 μg HeLa nuclear extract (Promega) or 10-20 μg His-fusion proteins over night at 4°C. Bound proteins were eluted by boiling in Laemmli buffer, resolved by a 12% SDS-PAGE and analyzed by Coomassie staining or immunoblot analysis.

To analyze protein interactions under different salt conditions, GST-GAS41 was incubated with HeLa nuclear extract. Sepharose was washed with 500 μl PBS followed by incubation with 500 μl PBS containing 0.14-1 M NaCl, for 45 min at 4°C. The remaining proteins were eluted, separated by 12% SDS-PAGE and subjected to immunoblot.

### Cell culture and co-immunoprecipitation

Cells were grown in DMEM (Invitrogen) supplemented with 10% fetal calf serum (Biochrom AG) at 37°C and 5% CO_2_. For immunoprecipitation, 2.5 × 10^6 ^cells were co-transfected with pSG5-HA-GAS41 and either p3xFLAG-CMV10-RAP30 or p3xFLAG-CMV10. As control pSG5 was co-transfected with p3xFLAG-CMV10-RAP30 or p3xFLAG-CMV10, respectively. Cells were disrupted in lysis buffer (150 mM NaCl, 50 mM Tris/HCl pH 8.0, 1% NP40, protease inhibitor cocktail (Roche)). For HA-co-immunoprecipitation we used HA-Tag IP/Co-IP kit (Pierce) according to the manufacturer's instructions. For FLAG-co-immunoprecipitation we used FLAG-tagged Protein Immunoprecipitation Kit (Sigma). 250 μg cell extract was incubated with 30 μl FLAG-M2 agarose over night at 4°C. After washing, bound proteins were eluted using 0.1 M glycine (pH 3.5) and subjected to 15% SDS-PAGE and immunoblot analysis. For nuclear extracts HeLa cells were lysed as described previously [15]. Lysates were subjected to immunoprecipitation using polyclonal GAS41 antibodies [15], rabbit IgG isotype control (Invitrogen), and protein A-sepharose (GE Healthcare). Precipitated protein were eluted by 1× Laemmli and subjected to 15% SDS-PAGE and immunoblot analysis.

## Authors' contributions

SH, NCH and NS carried out the experiments. AR participated in the design of the study. SH and NCH drafted the manuscript. EM conceived of the study, participated in its design and coordination and drafted the manuscript. All authors read and approved the final manuscript.
